# The Woman Workers' World

**Published:** 1918-07-20

**Authors:** 


### THE WOMAN WORKER'S WORLD.

Dr. Truby King's course of six lectures at the Marlborough School of Mothercraft, Trebovir Road, Earl's Court, is being well attended. The main point of the first was that cardinal article of his creed, that much of our infant mortality, directly due to dangers attending bottle-feeding, is indirectly the result of a long-prevalent idea that babies require food once in two or two and a half hours, and in the middle of the night. This idea, he thinks, crept into this country about 1870. Artificial food became common, and as cow's milk was supposed stronger, babies were filled up with large and frequent quantities of weak stuff which dilated the stomach and caused crying that might be thought due to hunger. Gradually the mother was required to feed with the same frequency, causing her to become a veritable slave to the child, and creating a natural reluctance to nurse for long or to bring up many successive children. Dr. King's discourses on population are always illuminating, his description of Chicago as the biggest city in *Poland*, containing 350,000 Poles, and of the preponderation of births among the less desirable elements of the population are poignant. Of 6,000 graduates of Cornell, etc., he calculates that in 200 years only 800 male descendants will remain.

Dr. King's studies in the formation of teeth are of great interest at a time when dentistry for our soldiers has proved so important a matter. Since at two months of pregnancy twenty little cells are beginning to form twenty teeth, and that immediately after birth the second teeth begin to form, it follows that upon proper food, fresh air, sleep, and exercise of the mother the child's comfort in this respect will depend. Actually before birth the little teeth with enamel and ivory are complete in the gums, waiting their time.

